# Use of Proton Pump Inhibitors Among Patients With Alcohol‐Related Cirrhosis—A Danish Nationwide Cohort Study

**DOI:** 10.1111/liv.70061

**Published:** 2025-03-11

**Authors:** Marine Sølling Ramsing, Morten Daniel Jensen, Peter Jepsen

**Affiliations:** ^1^ Department of Hepatology and Gastroenterology Aarhus University Hospital Aarhus Denmark; ^2^ Department of Clinical Epidemiology Aarhus University Hospital Aarhus Denmark

**Keywords:** alcohol‐related cirrhosis, epidemiology, nationwide cohort‐study, predictors, prevalence, proton pump inhibitors

## Abstract

**Background:**

Use of proton pump inhibitors (PPIs) may have adverse effects in patients with alcohol‐related cirrhosis (ALD cirrhosis), but PPIs continue to be used by many patients.

**Aims:**

We aimed to describe the prevalence and incidence of PPI use from filled prescriptions among patients with ALD cirrhosis and to identify predictors of PPI initiation after ALD cirrhosis diagnosis.

**Methods:**

We used Danish nationwide healthcare registries to investigate PPI use among patients diagnosed with ALD cirrhosis from 1997 to 2022. We used multivariable Cox regression to identify predictors of PPI initiation.

**Results:**

We identified 41 263 patients diagnosed with ALD cirrhosis in 1997–2022. In this cohort, the prevalence of PPI use rose to 40% in 2016 and plateaued at this level through 2022. Considering time since diagnosis, 26% were using PPI at the diagnosis of ALD cirrhosis, and the prevalence peaked at 38% 3 months later. Among PPI users, 79% used more than 30 defined daily doses per year on average during the follow‐up. Patients older than 50 years were more likely than younger patients to initiate PPI treatment.

**Conclusion:**

The use of PPIs continues to be prevalent among patients with ALD cirrhosis, with 40% of all patients using PPIs in 2022. Within the first 3 months after diagnosis, 38% of all patients were using PPIs. Our results provide essential background information for future RCTs on the risks and benefits of prescribing or deprescribing PPIs.

**Summary**

- PPIs may have adverse effects in patients with cirrhosis of the liver, but in the last two decades, studies have shown conflicting results.
- Our aim was to calculate the prevalence and the incidence of proton pump inhibitor use among all patients with cirrhosis in Denmark during this period.
- We showed that 40% of all patients with cirrhosis used PPIs and that many patients initiated treatment after they were diagnosed with cirrhosis.

**Abbreviations**
ALD: alcohol‐related cirrhosis
ATC: Anatomical Therapeutic Chemical
CI: confidence interval
DDD: defined daily doses
DNPR: The Danish National Prescription Registry
GERD: gastroesophageal reflux disease
HR: hazard rate
ICD‐10: International Classification of Diseases, 10th revision
NCSP: Nomesco Classification of Surgical Procedures,
NPR: The Danish National Patient Register
NSAID: non‐steroidal anti‐inflammatory drugs

## 1 Introduction

Patients with cirrhosis are vulnerable to debilitating and potentially deadly infections [1, 2]. The use of proton pump inhibitors (PPIs) may increase the risk of spontaneous bacterial peritonitis, *Clostridioides difficile* infections, and infections in general; however, the results are conflicting [3, 4, 5, 6, 7, 8]. Adding to this risk is the comparatively higher serum concentration of PPIs because of compromised hepatic elimination [9]. For these reasons, caution is recommended when prescribing PPIs to patients with alcohol‐related cirrhosis (ALD cirrhosis) [9, 10, 11]. Even so, PPIs are widely used among patients with ALD cirrhosis for acid‐related disorders and for prophylaxis when using non‐steroidal anti‐inflammatory drugs (NSAIDs). However, indications for PPI initiation are often unclear, causing concerns about overprescription [12, 13, 14].

While worries are increasing, it remains unknown if the prevalence of PPI use is increasing too. Several studies have reported the cross‐sectional prevalence of PPI use in smaller cohorts of cirrhotic patients, but none have examined the prevalence of PPI use in nationwide cohorts of patients with ALD cirrhosis [5, 9, 12]. The primary aim of this descriptive study was to calculate the prevalence and the cumulative incidence of PPI use among patients with ALD cirrhosis in the last two decades, where the studies of PPI use and cirrhosis complications have shown conflicting results (Table S1). Our secondary aim was to identify predictors of PPI use to better understand which patients initiate PPI treatment.

## 2 Patients and Methods

### 2.1 Data Sources

This nationwide cohort study was conducted using data from Danish healthcare registries. In Denmark, a unique personal identifier is given at birth or immigration to all citizens, and it enables individual‐level linkage of data from separate registries [15].

Patients with ALD cirrhosis were identified using The Danish National Patient Register (NPR). The NPR contains dates and discharge diagnoses from all hospital admissions and outpatient contacts in Denmark since 1995 [16, 17], coded in accordance with the International Classification of Diseases, 10th revision (ICD‐10) system. Public healthcare is fully tax‐financed and therefore free of charge at the point of use for all citizens in Denmark. Further, the data include surgical or procedural codes related to a given contact with the healthcare system, coded with the Nomesco Classification of Surgical Procedures (NCSP).

The Danish Civil Registration System was used to identify dates of birth, death and emigration [18, 19]. The Danish National Prescription Registry (DNPR) was used to extract dates and pack sizes of PPI and NSAID prescriptions, as well as spironolactone, furosemide, non‐selective beta blockers and lactulose, which we used to define the history of decompensation. The DNPR contains data from 1994 onwards and is considered complete since 1995 [20]. Purchase of prescription medications in Denmark is covered by a stepwise reimbursement system once an annual out‐of‐pocket payment has been reached. The DNPR does not account for over‐the‐counter sales of PPIs, but < 3% of all defined daily doses (DDDs) of PPIs are sold over the counter in Denmark [21].

### 2.2 Study Population

All patients diagnosed with ALD cirrhosis for the first time between 1 January 1997 and 31 December 2022 were identified in the NPR. Thus, patients with a diagnosis code for ALD cirrhosis before 1 January 1997 were excluded. To identify patients with ALD cirrhosis, we used the ICD‐10 diagnosis codes for alcohol‐related liver cirrhosis (K70.3), alcohol‐related liver insufficiency (K70.4), and additionally included patients with codes for unspecified cirrhosis (K74.6) if the patient previously had a diagnosis code wholly attributable to alcohol (codes shown in Table S2) [22]. Patients were followed from the date of the first diagnosis of ALD cirrhosis to death, emigration, or end of study on 31 December 2022. Patients younger than 18 years at the time of ALD cirrhosis diagnosis were excluded.

### 2.3 PPI Use

PPI use was defined as a prescription with the Anatomical Therapeutic Chemical (ATC) code A02BCx and considered to begin on the date the prescription was filled. The patient was considered a PPI user for two times the prescribed pack size, which left a ‘grace period’ at the end. If the patient filled a new prescription while being considered a PPI user, the patient would remain a PPI user. For each patient, we calculated the yearly filled DDD of PPIs. The DDD is the assumed average maintenance dose per day for a drug used for its main indication [23].

### 2.4 Predictors of PPI Initiation

At all times during the follow‐up, patients were characterised by a number of potential predictors. Many of these predictors were time‐dependent, and for each of them, Table S2 defines the event(s) that caused a change in the variable. The exposure to a potential predictor was set to begin on the first date of a hospitalisation coded with any of the relevant diagnosis codes or surgical procedure codes. Indications for PPI use were: gastroesophageal reflux disease (GERD), peptic ulcer, and prior NSAID use (defined using ATC codes, in the same manner as PPI use). We further included a history of decompensation as a potential predictor of PPI initiation. A history of decompensation was defined as having one of the following: a diagnosis code for ascites, spontaneous bacterial peritonitis, bleeding from oesophageal or gastric varices or hepatorenal syndrome (note, hepatic encephalopathy does not have an ICD‐10 diagnosis code); an ATC code for spironolactone, furosemide, non‐selective beta‐blockers or lactulose or a surgical procedure code for drainage of ascites fluid, treatment of variceal bleeding or insertion of a transjugular intrahepatic portosystemic shunt. History of decompensation began 180 days after the earliest of the above decompensation events. Predictors also included the area in which the patient resided at the time of cirrhosis diagnosis, divided into five regions: (1) Capital Region of Denmark, (2) Region Zealand, (3) Region of Southern Denmark, (4) Central Denmark Region and (5) North Denmark Region. All codes are shown in Table S2.

### 2.5 Statistical Analysis

#### 2.5.1 Prevalence of PPI Use

**FIGURE 1:**
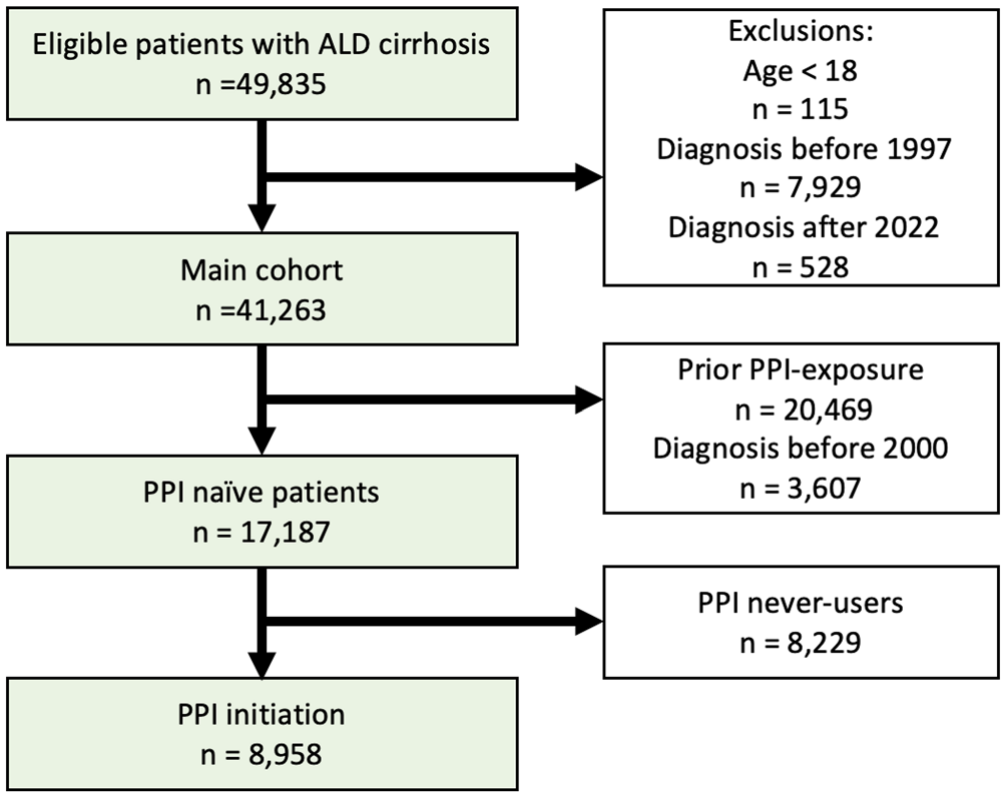
Flow chart of the main cohort and the PPI‐naïve subcohort that included the PPI initiators. The group that initiated PPI treatment was compared to all PPI‐naïve patients.

Analyses of prevalence included all patients diagnosed with ALD cirrhosis, between 1 January 1997 and 31 December 2022 (Figure 1, ‘Main cohort’).

##### 2.5.1.1 Prevalence

For each year, on 1 January, we computed the prevalence of PPI use as the number of patients who were using PPIs divided by the total number of patients on that date. Furthermore, we calculated the prevalence of PPI use based on time since cirrhosis diagnosis, on each day since diagnosis. We did not present prevalence estimates for 1997, 1998 and 1999 because the cohort was small in those early years and consisted exclusively of newly diagnosed patients (Figure S1).

##### 2.5.1.2 Quantitative Analysis of Annual DDD of PPI

For each PPI user, we calculated the average annual number of pills as the total number of pills from filled prescriptions during the follow‐up divided by the patient's follow‐up time from diagnosis. Similarly, we calculated the average annual dose of PPIs as the total dose divided by follow‐up time and then converted to DDD using the WHO's definition [23].

##### 2.5.1.3 Sensitivity Analysis of the Grace Period

To examine if the grace period caused us to overestimate the true prevalence of PPI use, we additionally computed the prevalence with no grace period and with a double‐length grace period. We further computed the proportion of patients who were or had been PPI users within the last 6 months and the prevalence of PPI users supposing patients took two pills per day.

#### 2.5.2 Initiation of PPI Use

We examined the initiation of PPI use after diagnosis of ALD cirrhosis including predictors of initiation. These analyses included only patients diagnosed with ALD cirrhosis after 1 January 2000, to ensure all included patients had at least 5 years without any filled PPI prescriptions prior to ALD cirrhosis diagnosis, and follow‐up ended on 31 December 2022 (Figure 1, ‘PPI naïve patients’).

##### 2.5.2.1 Cumulative Incidence

The cumulative incidence function was used to estimate the probability of becoming a first‐time PPI initiator with respect to time since ALD cirrhosis diagnosis while considering death as a competing event [24].

##### 2.5.2.2 Predictors

Cox proportional hazards regression was used to estimate the association between a candidate predictor and PPI initiation. The predictors included age, sex, region, history of decompensation, GERD, peptic ulcer and NSAID use. All variables were included in both a univariable and a multivariable analysis.

##### 2.5.2.3 Proportion of PPI Users Without a Detectable PPI Indication

We calculated the proportion of patients who initiated PPI treatment without any diagnosis of GERD, peptic ulcer disease or active NSAID prescriptions at the time of their first PPI prescription.

## 3 Results

We identified 41 263 patients with ALD cirrhosis between 1997 and 2022 (Figure 1). At the time of ALD cirrhosis diagnosis, 10 823 patients (26%) were using PPIs, and PPI users were slightly older than nonusers (median age 60 years vs. 57 years). More PPI users had a history of decompensation (44.1% vs. 27.3%), GERD (19.9% vs. 5.2%), peptic ulcer (22.4% vs. 9.1%), used NSAIDs (45.4% vs. 27.8%) or were from outside the Capital Region (66.1% vs. 61.3%). PPI users and nonusers had the same low prevalence of hepatitis B or hepatitis C infection: 3.2% (Table 1).

**TABLE 1:** Characteristics at the date of ALD cirrhosis diagnosis of PPI users and non‐users.

Individuals, No. (%)	Non‐users	Users
Number of patients	30 440	10 823
Age at diagnosis in years, median (interquartile range [IQR])	57 (50–65)	60 (53–67)
Female sex, *N* (%)	9442 (31.0)	3580 (33.1)
Region, *N* (%) – Capital Region of Denmark – Region Zealand – Region of Southern Denmark – Central Denmark Region – North Denmark Region	11 774 (38.7) 4978 (16.4) 4997 (16.4) 6441 (21.2) 2250 (7.4)	3673 (33.9) 1867 (17.3) 2032 (18.8) 2425 (22.4) 826 (7.6)
History of decompensation, *N* (%)	8312 (27.3)	4777 (44.1)
GERD, *N* (%)	1575 (5.2)	2154 (19.9)
Peptic ulcer disease, *N* (%)	2785 (9.1)	2422 (22.4)
NSAID, *N* (%)	8470 (27.8)	4919 (45.4)
Chronic hepatitis (HBV or HBC)	977 (3.2)	347 (3.2)

### 3.1 Prevalence of PPI Use

#### 3.1.1 Prevalence

**FIGURE 2:**
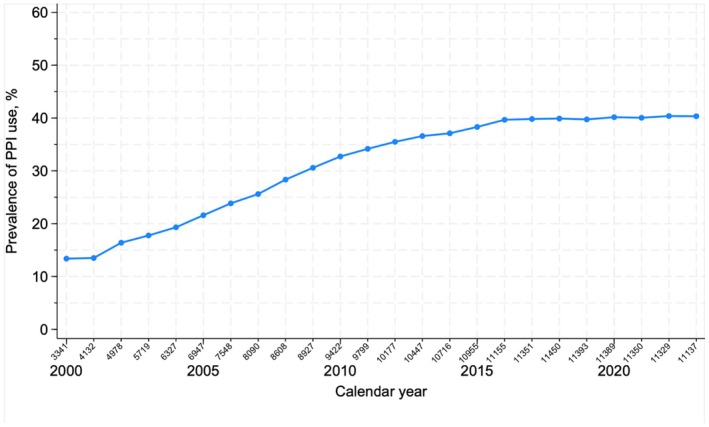
Prevalence of PPI use after diagnosis among patients with ALD cirrhosis in January every calendar year. We included patients whose first diagnosis of ALD cirrhosis was after 1 January 1997. Therefore, the cohort was small in 1997, 1998 and 1999 and consisted of newly diagnosed patients. From 2000, the cohort was a mix of newly diagnosed and patients surviving with cirrhosis in the later years.

**FIGURE 3:**
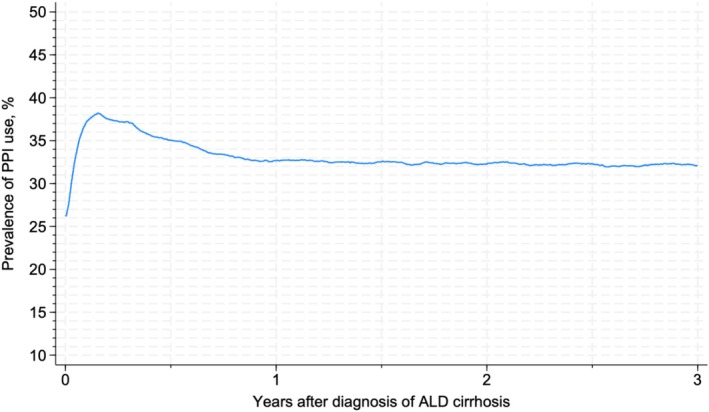
Prevalence of PPI use among patients with ALD cirrhosis based on years since cirrhosis diagnosis.

The prevalence of PPI use rose from 13.4% (95% CI: 12.2%–14.5%) in 2000 to 39.7% (95% CI: 38.1%–40.0%) in 2016 and plateaued at this level until 2022 (Figure 2). The prevalence of PPI use peaked at 38.2% (95% CI: 37.7%–38.7%) within the first 3 months after a diagnosis of ALD cirrhosis, followed by a drop to 32.7% (95% CI: 32.2%–33.2%) at 1 year after diagnosis, after which the prevalence remained stable (Figure 3).

#### 3.1.2 Quantitative Analysis of Annual DDD of PPIs

**FIGURE 4:**
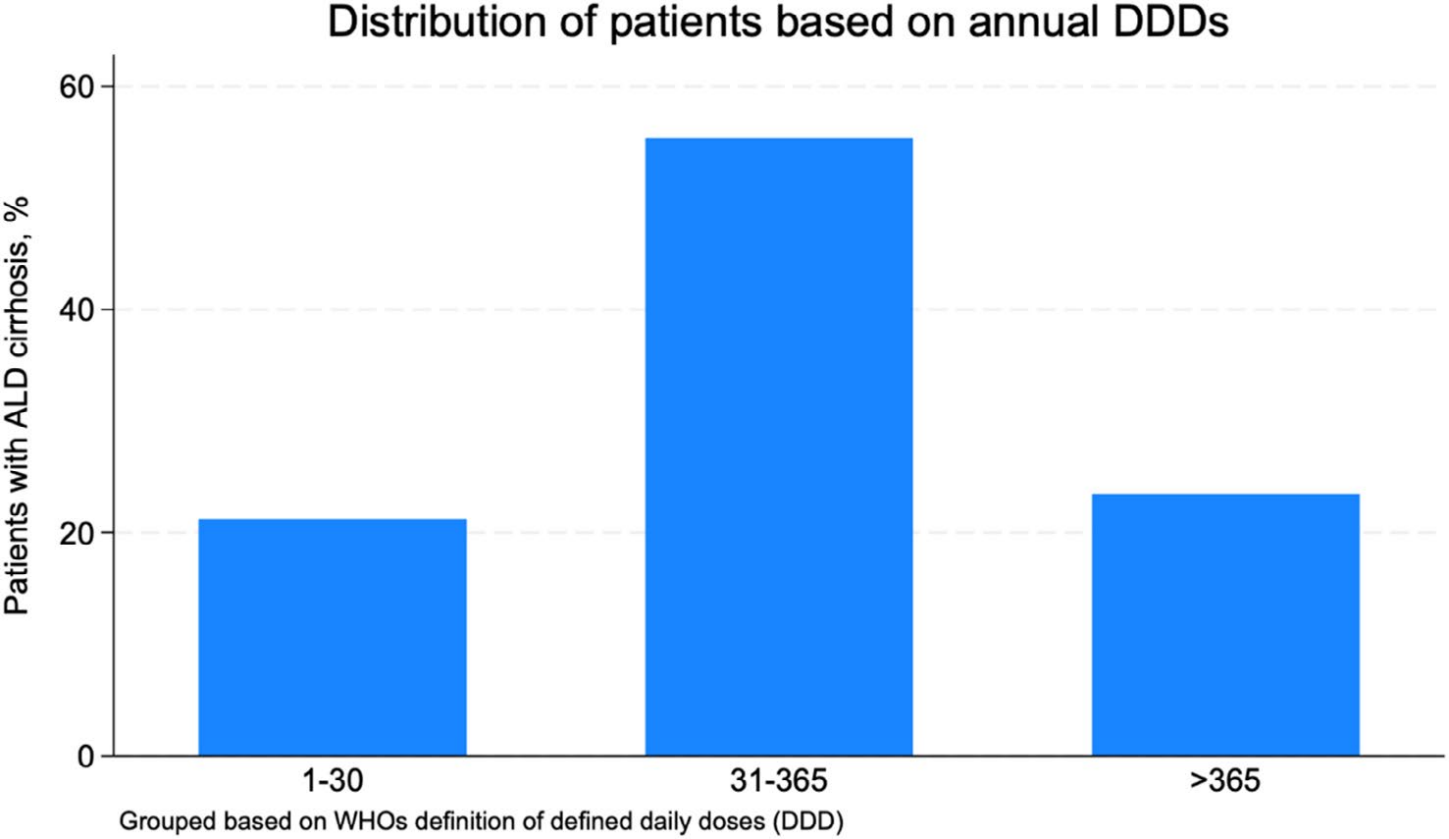
Distribution of PPI users based on annual defined daily doses (DDDs) of PPIs. (First) from 1 to 30 DDDs of PPIs filled per year. (Second) from 31 to 365 DDDs filled per year. (Third) more than 365 DDDs of PPIs filled per year.

Among patients who used PPIs after diagnosis, 78.8% filled more than 30 DDD of PPIs on average each year, and 23.5% filled more than 365 DDD each year (Figure 4). The median number of pills from filled prescriptions was 163 pills per patient per year (interquartile range [IQR]: 38–329) among PPI users. The median number of DDD was 170 per patient per year (IQR: 41‐348).

#### 3.1.3 Sensitivity Analysis of Grace Period

The prevalence of PPI use reached a plateau in 2016 independently of how we defined the grace period. With a grace period equal to the pack size (i.e., considered user for two times the pack size) a plateau was reached at 40%. When discarding the grace period, the plateau was reached at 34%, and when doubling the grace period, the plateau was reached at 43%. With a grace period of 6 months, a plateau was reached at 44% (i.e., 44% were or had been PPI users within the last 6 months). If patients were only considered PPI users for the number of days corresponding to half the pack size, a plateau was reached at 21% (i.e., 21% were PPI users if PPI dose was assumed to be two pills per day).

### 3.2 Initiation of PPI Use

#### 3.2.1 Cumulative Incidence

Among the 17 187 PPI‐naïve patients diagnosed after 1 January 2000, 8958 patients initiated PPI treatment after they were diagnosed with ALD cirrhosis (Table S3), many of them shortly after. Specifically, the cumulative incidence of PPI initiation from the time of ALD cirrhosis diagnosis was 22.0% (95% CI: 21.4%–22.6%) at 3 months, 25.7% (95% CI: 25.0%–26.3%) at 6 months, 29.7% (95% CI: 29.0%–30.4%) at 1 year and 39.5% (95% CI: 38.8%–40.3%) at 3 years (Figure S2).

#### 3.2.2 Predictors

Patients aged 50–59 years were more likely to initiate PPI treatment than younger patients aged < 50, hazard rate (HR) 1.12 (95% confidence interval (CI): 1.05–1.19). Similarly, older patients aged 60–69 years and 70–79 years were more likely to initiate PPI treatment than patients aged < 50, HR 1.18 (95% CI: 1.11–1.26) and 1.17 (95% CI: 1.09–1.26) respectively.

Patients living in the Region of Southern Denmark were less likely to initiate PPI treatment after diagnosis, HR 0.87 (95% CI: 0.82–0.93) compared with patients living in the Capital Region of Denmark. A history of decompensation predicted PPI initiation (HR: 1.16 [95% CI: 1.11–1.22]) as did GERD (HR: 3.40 [95% CI: 3.12–3.71]), peptic ulcer (HR: 5.05 [95% CI: 4.75–5.38]) and NSAID use (HR: 1.26 [95% CI: 1.21–1.32]) (Table 2). Among the 8958 PPI‐naïve patients who initiated PPI treatment after diagnosis, 55.3% did not have GERD, peptic ulcer or an active NSAID prescription.

**TABLE 2:** Predictors of PPI initiation (*N* = 8958) among the patients (*N* = 17 187) who were diagnosed with ALD cirrhosis after 2000 and had not previously used PPI.

Hazard ratio (95% CI)	Univariable analysis	Multivariable analysis (adjusted)
Age – < 50 – 50–59 – 60–69 – ≥ 70	Ref 1.16 (1.10–1.24) 1.25 (1.18–1.33) 1.24 (1.16–1.34)	Ref 1.12 (1.05–1.19) 1.18 (1.11–1.26) 1.17 (1.09–1.26)
Women	0.97 (0.93–1.01)	1.00 (0.96–1.05)
Region – Capital Region of Denmark – Region Zealand – Region of Southern Denmark – Central Denmark Region – North Denmark Region	Ref 1.05 (0.99–1.12) 0.93 (0.87–0.99) 0.99 (0.94–1.05) 1.03 (0.95–1.12)	Ref. 1.04 (0.97–1.10) 0.87 (0.82–0.93) 1.00 (0.94–1.05) 1.00 (0.92–1.09)
History of decompensation	1.27 (1.21–1.33)	1.16 (1.11–1.22)
GERD	4.49 (4.13–4.88)	3.40 (3.12–3.71)
Peptic ulcer disease	5.83 (5.49–6.20)	5.05 (4.75–5.38)
NSAID	1.28 (1.23–1.34)	1.26 (1.21–1.32)

## 4 Discussion

We identified all patients with ALD cirrhosis in Denmark and described the prevalence, incidence and predictors of PPI use: The prevalence rose to around 40% and plateaued at this level from 2016 to the end of 2022. Among PPI users, 78.8% filled more than 30 DDDs of PPIs per year, and 23.5% filled more than 365 DDDs per year. Many patients were put on PPIs after cirrhosis diagnosis, the prevalence peaked at 38.2% within 3 months after diagnosis, and the cumulative incidence was 29.7% after the first year. Patients were more likely to initiate PPI treatment if they had an indication such as GERD, peptic ulcer disease or were using NSAIDs, but 55.3% of the patients who initiated PPI treatment did not have any of those indications. We found that patients living in the Region of Southern Denmark were less likely to initiate PPI treatment compared to patients living in the Capital Region of Denmark. We could not determine whether this difference resulted from regional variation in unmeasured patient characteristics or from regional variation in physicians' willingness to prescribe PPIs.

This population‐based study included all patients with a hospital diagnosis of ALD cirrhosis in Denmark, and all Danish citizens can be followed to death or emigration; hence, selection bias in cohort studies like ours is essentially eliminated. Our results are generalisable to countries with comparable healthcare systems, prescription practices and reimbursement of prescribed medications.

We identified patients with ALD cirrhosis in the NPR. The positive predictive value of a cirrhosis diagnosis code in the NPR is high, estimated at 85.4% in a study from 1997 (95% CI: 79.8–89.6) [25]. In 2011, a study of the NPR found that a diagnosis code of moderate/severe liver disease had a positive predictive value of 100% (95% CI: 92.9–100) compared to the patients' discharge notes [26]. The completeness is considered high as well, as health care is fully tax‐financed in Denmark, and all patients with suspected severe liver disease are referred from the general practitioner to a hospital for diagnostic workup and treatment. Therefore, any possible misclassification is minor and unlikely to have biased our results.

The DNPR was used to identify all PPI prescriptions. Valid, high‐quality data were available since 1994, and it is considered complete since 1995 [20]. Although the actual use of medicine is unknown, the patient filled the prescription and had the medicine in their possession on the date defined as the date of PPI initiation. Unfortunately, the indications listed for the PPI prescriptions are incomplete, as well as the intended duration and dosage. We therefore presumed that patients took one pill each day, but the prescription could also have been intended as ‘use as necessary’. To further investigate the magnitude of this uncertainty, we conducted a sensitivity analysis with five different interpretations of the length of PPI use. Even when we made considerable changes in our definition of PPI use, the estimates prevalence of PPI use remained around 34%–44%. Supposing all patients took two pills daily, pack size representing half the length of use, the prevalence would be lower, around 21%. However, this would be unlikely in the majority of patients, since they already receive high doses of PPIs and a double dose would be improbable (the DDD of pantoprazole is 40 mg).

We found the prevalence of PPI use among patients with ALD cirrhosis in Denmark to reach a plateau at 40% from 2016 to 2022. This is in line with previous studies, which found that PPIs are widely prescribed to patients with severe liver disease and cirrhosis, ranging from 40% to 78% depending on the population and the definition of PPI use [5, 12, 13]. By comparison, in the adult Danish population, only 7% were using PPIs in 2014 [27]. PPIs are predominantly prescribed in primary care settings (94%), where physicians may be less aware of the risks associated with PPI use among patients with cirrhosis [28]. The need to de‐prescribe could have been overlooked or could have been unsuccessful because of rebound acid hypersecretion [29]. A study showed that 71% of long‐term PPI users with cirrhosis who discontinue PPI treatment for ≥ 90 days return to PPI use [30].

Established indications for PPI initiation include GERD, peptic ulcer disease and use of NSAIDs, and patients were more likely to initiate PPI treatment if they had one of these diagnoses or used NSAIDs. GERD is a common and unspecific symptom; consequently, we expect some patients without the ICD‐10 code to have GERD symptoms and get relevant treatment. Still, more than half of the patients who initiated PPI treatment had no detectable indication for PPI use in our data. This is in line with several previous studies: A Swedish study from 2008, of 128 patients with liver cirrhosis of any cause, examined the indications behind the many PPI prescriptions and found that 63% did not have an adequate indication for PPI use [12]. Similarly, a recent Romanian study reviewed the hospital records of PPI users with cirrhosis of any aetiology, and 47% of patients had no relevant indication for PPIs [31]. In both studies, indications were similar to ours, including GERD, peptic ulcer and NSAID use.

Studies on PPI use and the association with cirrhosis complications and bone fractures have shown conflicting results in the last two decades [4, 5, 7, 8, 32, 33, 34, 35]. These studies have mainly been observational, efficient in showing a potential association between PPIs and cirrhosis complications, but with the risk of uncontrolled confounding (Table S1). Causation should ideally be deduced from well‐designed RCTs. Another important limitation of previous studies is the fact that PPI use may vary over time, so defining PPI users only at inclusion may result in misclassification and underestimation of effects (Table S1). Our nationwide study considered changes in PPI use during the follow‐up and showed that PPIs continue to be prescribed frequently. Future studies evaluating PPI treatment among patients with ALD cirrhosis should account for the morbidity associated with PPI use. Predictors of PPI initiation included a history of decompensation, as well as GERD and peptic ulcer. For now, clinicians should be cautious when prescribing PPIs to patients with ALD cirrhosis and always consider de‐prescribing [29]. Our results provide essential background information for future RCTs on the risks and benefits of prescribing and deprescribing PPI in patients with ALD cirrhosis.

## 5 Conclusion

Our nationwide cohort study of the prevalence of PPI use from filled prescriptions among patients with ALD cirrhosis showed that 40% were using PPIs in 2022. The prevalence has remained stable, although the controversy about PPIs and their association with cirrhosis complications persists. Reassuringly, the survival for Danish patients with ALD cirrhosis has improved despite the increasing prevalence of PPI use: The relative risk of 1‐year all‐cause mortality in 2014–2018 versus 1994–1998 was 0.83 (95% CI: 0.78–0.87) [36]. Regardless of the continuing debate on PPI use and the risk of cirrhosis complications, many patients initiate PPI treatment after they are diagnosed with cirrhosis.

## Author Contributions

All authors made a significant contribution to the work reported. **Marine Søllinag Ramsing:** writing – original draft (lead), formal analysis (lead). **Morten Daniel Jensen:** conceptualization (supporting), formal analysis (supporting), interpretation (supporting), writing – review and editing (equal). **Peter Jepsen:** conceptualization (lead), acquisition of data (lead), interpretation (lead), writing – review and editing (equal). All authors approved the final version of the article, including the authorship list.

## Ethics Statement

The authors have nothing to report.

## Consent

This nationwide registry‐based cohort study was conducted in accordance with Danish law. No individual consent was needed since the data from the Danish healthcare registries was pseudonymized.

## Conflicts of Interest

The authors declare no conflicts of interest.

## Supporting information


Data S1.

## Data Availability

This study was conducted using Danish healthcare registries. The Danish Health Data Authorities maintain the data sources, and access is restricted and only available through an application.
