# Supplementary material for: Use of Proton Pump Inhibitors Among Patients With Alcohol‐Related Cirrhosis—A Danish Nationwide Cohort Study
Source: Liver Int. 2025 Mar 11;45(4):e70061. doi: 10.1111/liv.70061 (PMC11894920; doi:10.1111/liv.70061)

Supplementary material

Supplementary Table S1: Studies of adverse events of PPI use among patients with liver cirrhosis

Study	Year	Patients	PPI use	Outcome	Method	Findings	Limitations
Gairing et al.[1]	2024	1,160 patients with MELD 11. Diagnosis based on histology, ultrasound, elastography, endoscopy, medical history or radiological images. Excluded if diagnosed with dementia or stroke.	PPI use, type and indication recorded at inclusion.	MHE diagnosed based on PHES score. OHE diagnosed according to the West Haven Criteria.	Follow-up. Multicenter.	PPI use was neither asso-ciated with the presence of MHE (OR: 1.07 (95% CI: 0.80–1.43) at baseline nor OHE development during follow-up sHR: 1.13 (95% CI: 0.81–1.59)	Observational study. Risk of unknown confounding. Retrospective collection of PPI data. Only PPI data at inclusion - not suitable to detect time dependent effect of PPI. Exclusion criteria varied at different centers. No nation-wide cohort.
Dam et al.[2]	2016	865 patients with cirrhosis and ascites. Post hoc analysis of data from the satavaptan-trials. Excluded if SBP episode 10 days before inclusion, and	PPI use, dosage and indications recorded every 4 weeks.	HE graded 1-4 according to the West Haven Criteria<br><br>SBP	Follow-up. Multicenter.	PPI use associated with OHE HR: 1.36 (95% CI, 1.01–1.84) and SBP HR: 1.72 (95% CI: 1.10–2.69).	Observational study. Risk of unknown confounding. Risk of uncontrolled confounding by MHE and of

		previous HE in the analyses of HE.					time-dependent confounding
Nardelli et al.[3]	2019	310 patients with cirrhosis. Diagnosis based on clinical, biochemical, and radiological signs. Exclusion if OHE, alcohol/psychoactive drugs, dementia or unrelated neurological disease	PPI use, dosage and duration recorded at admission or from medication lists. "PPI users" if treated 4 weeks prior to admission	MHE diagnosed by PHES test ≤ -4<br><br>OHE diagnosed by the West Haven Criteria.<br><br>Overall survival.	Follow-up. Cross-sectional study with respect to MHE. Single center	Higher prevalence of MHE among PPI users than in non-users (62% versus 29%; P < 0.001)<br><br>Incidence of HE higher in PPI users than in nonusers (64% versus 25%, P < 0.001) with risk of death or liver transplantation as competing events.<br><br>Overall survival lower in PPI users than in nonusers (41% versus 81%, P < 0.001).	Observational study. Risk of unknown confounding. Relatively small cohort. Retrospective collection of PPI data.<br>Only PPI data at inclusion - not suitable to detect time dependent effect of PPI. Risk of differentiated misclassification as the outcome MHE was measured at the same time as the exposure PPI. No nation-wide cohort.
Terg et al.[4]	2015	519 patients with decompensated cirrhosis. Diagnosis of cirrhosis established either with a liver biopsy or by a	PPI use in the previous 3 months recorded at inclusion.	SBP diagnosed by cell count in ascitic fluid ≥250 cells/mm^3	Follow-up. Multicenter	No significant difference in the rate of PPI consumption between patients with and without	Observational study. Risk of unknown confounding. Risk of residual confounding.

		combination of physical, endoscopic, laboratory and ultrasonographic findings.<br><br>Excluded if active gastrointestinal bleeding, antibiotic treatment in the previous 2 weeks including quinolone or rifaximin prophylaxis or if HIV-positive or immunosuppressive therapy.		Infections: Spontaneous bacteremia diagnosed by positive blood culture. Urinary infection diagnosed by positive urine culture. Other infections diagnosed according to conventional criteria.		SBP: 46.3% (44 out of 95 patients) vs. 42% (121 out of 289 patients).<br><br>No significant difference in the rate of PPI consumption between infected and non-infected patients (44.3% vs. 42.8%)	Risk of confounding due to SBP being related to HE and PPI.<br><br>Relatively small cohort: 95 patients with SBP.<br><br>Risk of misclassification because of, information based on memory.
Labenz[5]	2020	1,795 patients with liver cirrhosis and fractures compared to 10,235 patients without fractures.	PPI use overall; at least one prescription from a general practitioner. The cumulative PPI dose 5 years prior to the index date.	Any bone fractures diagnosis, coded with ICD-10.	Nested case-control study	PPI use associated with bone fractures OR: 1.34 (95% CI: 1.20–1.51). Dose dependent effect observed.	Observational study. Risk of unknown confounding. Risk of residual confounding due to missing details on liver function, liver disease severity and MHE. Risk of misclassification due to

							undercoding or miscoding of diagnosis codes.

CI: Confidense interval, HE: Hepatic encephalopathy, HR: Hazard ratio, MHE: Minimal hepatic encephalopathy, OHE: Overt hepatic encephalopathy, OR: Odds ratio, PPI: Proton pump inhibitors, SBP: Spontaneous bacterial peritonitis, sHR: Subdistribution hazard ratio

1. Gairing SJ, Mangini C, Zarantonello L, et al. Proton pump inhibitor use and risk of hepatic encephalopathy: A multicentre study. *JHEP Rep*. Aug 2024;6(8):101104. doi:10.1016/j.jhepr.2024.101104
2. Dam G, Vilstrup H, Watson H, Jepsen P. Proton pump inhibitors as a risk factor for hepatic encephalopathy and spontaneous bacterial peritonitis in patients with cirrhosis with ascites. *Hepatology*. Oct 2016;64(4):1265-72. doi:10.1002/hep.28737
3. Nardelli S, Gioia S, Ridola L, Farcomeni A, Merli M, Riggio O. Proton Pump Inhibitors Are Associated With Minimal and Overt Hepatic Encephalopathy and Increased Mortality in Patients With Cirrhosis. *Hepatology*. Aug 2019;70(2):640-649. doi:10.1002/hep.30304
4. Terg R, Casciato P, Garbe C, et al. Proton pump inhibitor therapy does not increase the incidence of spontaneous bacterial peritonitis in cirrhosis: a multicenter prospective study. *J Hepatol*. May 2015;62(5):1056-60. doi:10.1016/j.jhep.2014.11.036
5. Labenz C, Wörns MA, Adarkwah CC, Galle PR, Schattenberg JM, Kostev K. Proton pump inhibitors increase risk of bone fractures in men with cirrhosis: a population-based study. *Aliment Pharmacol Ther*. Sep 2020;52(6):1042-1050. doi:10.1111/apt.16008

Supplementary Table S2. Predictors of PPI initiation or discontinuation, definitions, and codes.

Covariate	Measure	Definition	Codes
Age	Categorical, time varying, current age	< 50 years<br>50–59 years<br>60–69 years<br>≥ 70 years	
Sex	Binary, constant	Male/female	
Calendar year	Binary, time varying	Before and after 1 January 2005 and 1 January 2010	

Socioeconomic variables

Education	Categorical, constant	Highest completed education in 2019	
Employment status	Categorical, time varying	Employment status in November the year before diagnosis	
Cohabitation status	Binary, time varying	Living together with a partner	
Region	Categorical, constant	The region in which the patient lived at the time of the first hospitalisation	

Cirrhosis

<table>
<tr><td rowspan="10">ALD cirrhosis</td><td rowspan="10">Binary
ICD-10 codes A and B diagnoses</td><td colspan="2">Cirrhosis due to alcohol</td><td>K70.3x,</td></tr>
<tr><td colspan="2">Liver failure due to alcohol</td><td>K70.4x</td></tr>
<tr><td rowspan="8">Specified or unspecified cirrhosis K74.6 + diagnosis codes wholly attributable to alcohol beforehand</td><td>Alcohol-induced pseudo-Cushing’s syndrome</td><td>E24.4</td></tr>
<tr><td>Mental and behavioural disorders due to use of alcohol</td><td>F10</td></tr>
<tr><td>Degeneration of nervous system due to alcohol</td><td>G31.2</td></tr>
<tr><td>Alcoholic polyneuropathy</td><td>G62.1</td></tr>
<tr><td>Alcoholic myopathy</td><td>G72.1</td></tr>
<tr><td>Alcoholic cardiomyopathy</td><td>I42.6</td></tr>
<tr><td>Alcoholic gastritis</td><td>K29.2</td></tr>
<tr><td>Alcoholic liver disease</td><td>K70</td></tr>
</table>

			Alcohol-induced chronic pancreatitis	K86.0
			Ethanol poisoning	T51.0
			Methanol poisoning	T51.1
			Toxic effect of alcohol, unspecified	T51.9
			Accidental poisoning by and exposure to alcohol	X45
			Intentional self-poisoning by and exposure to alcohol	X65
			Poisoning by and exposure to alcohol, undetermined intent	Y15
			Alcohol-induced acute pancreatitis	K85.2
			Excess alcohol blood levels	R78.0
			Evidence of alcohol involvement determined by blood alcohol level	Y90
			Evidence of alcohol involvement determined by level of intoxication	Y91

PPI use

PPI initiation	Binary<br>ATC codes	ATC-codes<br>- Omeprazole<br>- Pantoprazole<br>- Lansoprazole<br>- Esomeprazole<br>- Dexlansoprazole<br>- Dexrabeprazole<br>- Vonoprazan<br>- Tegoprazan<br>- Lansoprazole, combi<br>- Rabeprazole, combi	A02BCx
PPI discontinuation	Binary	No new filled prescription while the patient was considered a PPI user.	

NSAID use

NSAID initiation	Binary<br>ATC codes<br>Beginning on the date of first filled prescription.	Acetylsalicylic acids (ASA)	NO2BA<br>B01AC06
		ASA + corticosteroids	M01BA
		ASA + PPI	B01AC56
		NSAID + opioids<br>- ASA + codein<br>- Ibuprofen + codein<br>- Dexketoprofen + tramadol<br>- Celecoxib + tramadol<br>- ASA + oxycodon	 N02AJ02<br>N02AJ07<br>N02AJ08<br>N02AJ14<br>N02AJ16<br>N02AJ18
		NSAID<br>- Acetic acid derivates and related substances<br>- Oxicams<br>- Propionic acid derivates<br>- Fenmates<br>- Coxib<br>- Ditazole<br>- Carbasalate calcium<br>- Indobufen<br>- Aloxiprin<br>- Triflusal	 M01AB<br><br>M01AC<br>M01AE<br>M01AG<br>M01AH<br>B01AC01<br>B01AC08<br>B01AC10<br>B01AC15<br>B01AC17
		ASA + statins	C10BX01<br>C10BX02<br>C10BX04<br>C10BX05<br>C10BX06<br>C10BX07<br>C10BX08<br>C10BX12

Decompensation

Decompensation	Binary, beginning 180 days after the date of a cirrhosis decompensation event<br>ICD-10 codes	Ascites	R18x
		Spontaneous bacterial peritonitis	K658I
		Oesophageal variceal bleeding	I850
		Gastric variceal bleeding	I864A
		Hepatorenal syndrome	K767

	NCSP codes	Drainage of ascites fluid	KTJA10, JAL96
		Treatment of variceal bleeding	KJCA20, KJCA22, KJDA22, KJCA 32 KJCA35, KTPH10
		Transjugular intrahepatic portosystemic shunt	KPHW35A
	ATC codes	Spironolactone	C03DA01
		Furosemide	C03CA01
		Non-selective beta-blockers	C07AA05 C07AG02
		Lactulose	A06AD11

Indications for PPIs

GERD	Binary ICD-10 codes	Gastroesophageal reflux<br>Esophagitis (excl. abscess in oesophagus, K209A)<br>Barrets esophagus	K21<br>K20x<br><br>K22.7
	Binary NCSP codes	Gastroesophageal reflux	KJBC
Peptic ulcer disease	Binary ICD-10 codes	Ulcer in oesophagus<br>Acute/chronic ulcer<br>Duodenal ulcer<br>Gastroduodenal ulcer<br>Gastrointestinal ulcer	K22.1<br>K25<br>K26<br>K27<br>I28
	Binary NCSP codes	Gastroscopic<br>- Injection in ventricle<br>- Coagulation in ventricle<br>- Laser-operation in ventricle<br>Other hæmostasis in ventricle<br>Laporascopic suture of ventricle<br>Gastroscopic<br>- Injection in duodenum<br>- Coagulation in duodenum<br>- Laser operation in duodenum<br>Other hæmostasis in duodenum<br>Suture of duodenum<br>Laporascopic suture of duodenum	 KJDA32<br>KJDA35<br>KJDA38<br>KJDA42<br>KJDA60<br><br>KJDH15<br>KJDH18<br>KJDH22<br>KJDH25<br>KJDH70<br>KJDH71

Hepatitis

Hepatitis B and C	Binary<br>ICD-10 codes	Chronic viral hepatitis	B18x

Supplementary Table S3. Characteristics of PPI naïve patients on the date of ALD cirrhosis diagnosis

**Individuals, No. (%)**	**PPI-naïve patients**
Number of patients	17,187
Age at diagnosis in years, median (interquartile range [IQR])	59 (51–67)
Female sex, N (%)	5,208 (30.3)
Region, N (%)<br>- Capital Region of Denmark<br>- Region Zealand<br>- Region of Southern Denmark<br>- Central Denmark Region<br>- North Denmark Region	 6,656 (38.7)<br>2,831 (16.5)<br>2,853 (16.6)<br>3,569 (20.8)<br>1,278 (7.4)
History of decompensation, N (%)	4,078 (23.7)
GERD, N (%)	372 (2.2)
Peptic ulcer disease, N (%)	835 (4.9)
NSAID, N (%)	4,506 (26.2)

***Supplementary Figure S1*. Prevalence of cirrhosis during follow up based on calendar time.** Main cohort (blue), overall users (red), prior user/continuator (green), first-time user (PPI naïve at diagnosis) (yellow).

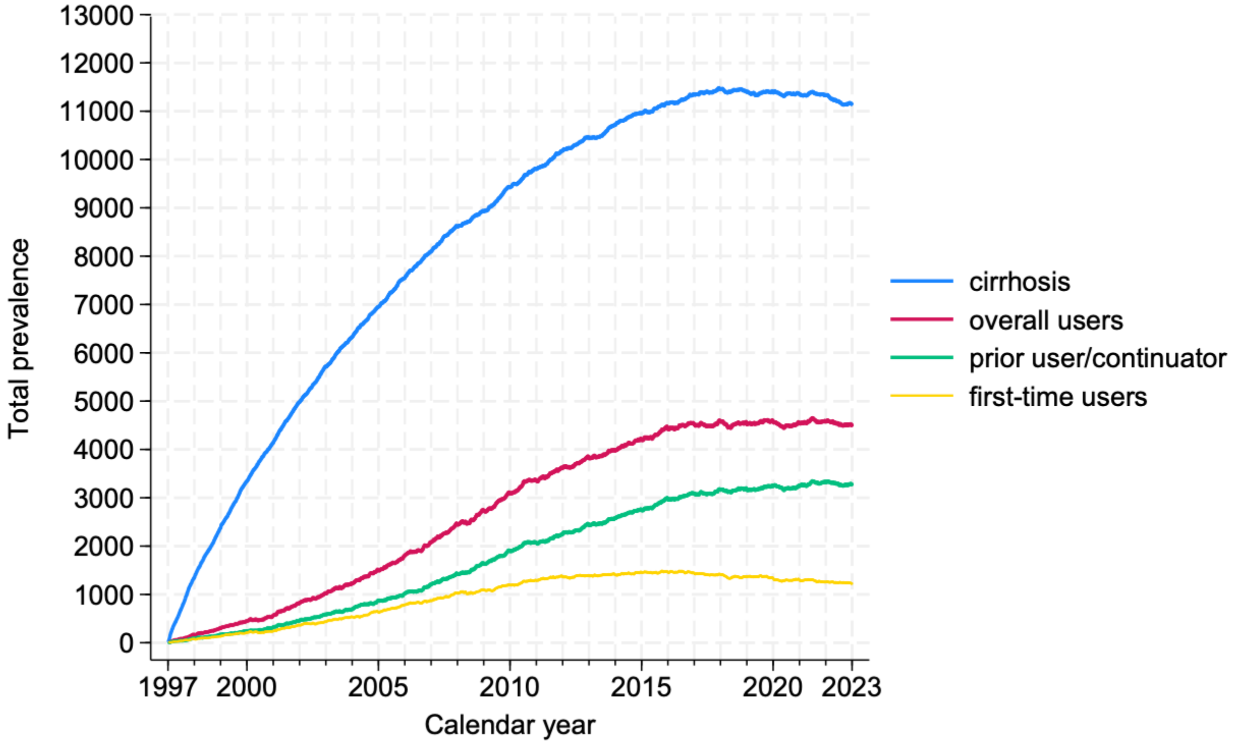

***Supplementary Figure S2*.** Cumulative proportion of new PPI users.

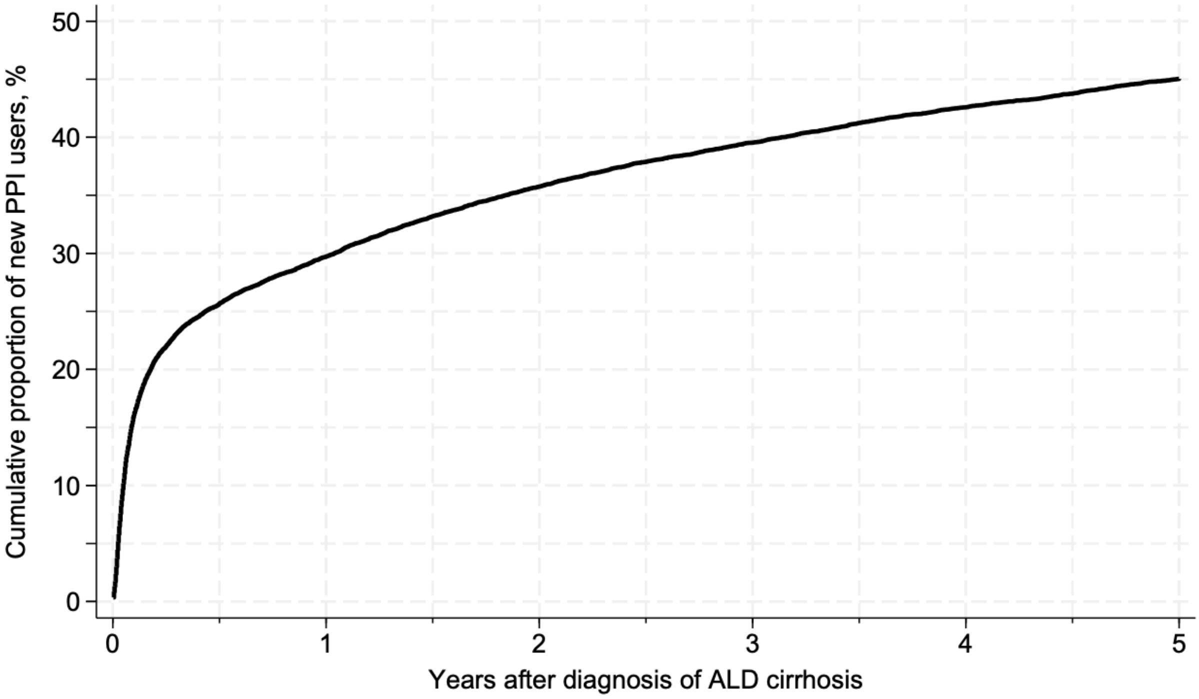

Supplement: Supplementary file 1 — Data S1. [file LIV-45-0-s001.pdf]
